# The pan-plastome of *Hemerocallis citrina* reveals new insights into the genetic diversity and cultivation history of an economically important food plant

**DOI:** 10.1186/s12870-023-04668-z

**Published:** 2024-01-11

**Authors:** Minlong Jia, Jie Wang, Dongmei Cao, Congrong Jiang, Wei Li, Luke R. Tembrock, Guoming Xing, Sen Li, Zhiqiang Wu

**Affiliations:** 1 College of Horticulture, Shanxi Agricultural University, Taiyuan, 030031 China; 2 Shenzhen Branch, Guangdong Laboratory of Lingnan Modern Agriculture, Key Laboratory of Synthetic Biology, Laboratory of the Ministry of Agriculture and Rural Affairs, Agricultural Genomics Institute at Shenzhen, Chinese Academy of Agricultural Sciences, Shenzhen, 518120 China; 3 College of Science, Health, Engineering and Education, Murdoch University, Perth, WA 6000-6999 Australia; 4 Kunpeng Institute of Modern Agriculture at Foshan, Shenzhen Branch, Guangdong Laboratory of Lingnan Modern Agriculture, Agricultural Genomics Institute at Shenzhen, Chinese Academy of Agricultural Sciences, Shenzhen, 518124 China; 5 Department of Agricultural Biology, Colorado State University, Fort Collins, CO 80525 USA

**Keywords:** *Hemerocallis citrina*, Panplastome, Phylogeny, Population structure

## Abstract

**Background:**

*Hemerocallis citrina* Baroni (Huang hua cai in Chinese) is a perennial herbaceous plant grown for its flower buds that are eaten fresh or dried and is known as the vegetarian three treasures. The nuclear genome of *H. citrina* has been reported, but the intraspecific variation of the plastome (plastid genome) has not yet been studied. Therefore, the panplastome of this species collected from diverse locations is reported here for the first time.

**Results:**

In this study, 65 *H. citrina* samples were resequenced, de novo assembled, and aligned with the published plastome of *H. citrina* to resolve the *H. citrina* panplastome. The sizes of the 65 newly assembled complete plastomes of *H. citrina* ranged from 156,048 bp to 156,263 bp, and the total GC content ranged from 37.31 to 37.34%. The structure of the complete plastomes showed a typical tetrameric structure, including a large single copy (LSC), a small single copy (SSC), and a pair of inverted repeat regions (IRA and IRB). Many nucleotide variants were identified between plastomes, among which the variants in the intergenic spacer region were the most abundant, with the highest number of variants concentrated in the LSC region. Based on the phylogenetic tree constructed using the ML method, population structure analysis, and principal component analysis (PCA), the panplastome data were subdivided into five genetic clusters. The C5 genetic cluster was mostly represented by samples from Qidong, Hunan Province, while samples from Shanxi and Shaanxi Provinces were classified into the C4 genetic cluster. The greatest genetic diversity was found in the C1 genetic cluster, and the greatest genetic distance between any two clusters was found between the C4 and C5 clusters.

**Conclusion:**

The resolution of the panplastome and the analysis of the population structure of *H. citrina* plastomes provide important data for future breeding projects and germplasm preservation.

**Supplementary Information:**

The online version contains supplementary material available at 10.1186/s12870-023-04668-z.

## Introduction

*Hemerocallis citrina* Baroni, commonly referred to as the citron daylily, is a perennial herbaceous plant found growing wild and cultivated in Asia, including China, Japan, and Korea. The *H. citrina* flower buds are highly prized as a flavourful vegetable in Asian cuisine. The cultivation history of *H. citrina* dates back 2500 years [1, 2]. In addition to their use as food, *H. citrina* flower buds have long been used medicinally, as mentioned in the “*Compendium of Materia Medica*” for promoting lactation [3] and in the Chinese Pharmacopoeia for improving sleep [4]. More recently, tests with *H. citrina* extracts have indicated therapeutic effects against certain human ailments, such as depression and diabetes [5–7]. Bioactive substances extracted from *H. citrina* have shown antimicrobial, antioxidant, and nitrite elimination activities [3]. Due to its rich nutritional and potential medicinal value, the demand for *H. citrina* is growing rapidly. The market value of *H. citrina* has been estimated at 1 billion US dollars for the last several years of production [8]. Therefore, the collection, preservation, and characterization of *H. citrina* germplasm and the development of new varieties are particularly important tasks. An essential step in studying germplasm and developing new cultivars is the completion of large-scale genomic analyses. At present, only a handful of studies have generated genomic data, including the generation of a draft nuclear genome at an impressive 3.77 GB [8]. Additionally, Hirota et al. previously inferred the evolutionary history of Japanese daylilies using plastomic and nuclear markers to study interspecific gene flow, but the study did not include samples from mainland Asia [9]. Given the lack of intraspecific studies on *H. citrina* and the impressive size of the nuclear genome, plastome sequencing efforts are currently well suited for characterizing genomic diversity in this economically important species.

Unlike the nuclear genome [10], the plastome contains no genes directly responsible for floral organ development; however, it is the control centre of the plastid, including the chloroplast, and as such is central to key metabolic processes in the plant cell, such as photosynthesis and the storage of starch. Because of the small size of the plastome and its essential function, it is found in high copy number in most living plant cells, making DNA extraction and sequencing highly efficient. Due to the conserved genomic structure and lack of recombination found in plastomes, complete genomic assembly is very accurate. Despite the conserved genomic structure, numerous regions within the plastome are evolving rapidly enough to resolve intraspecific lineages. Therefore, plastomes can be used in studies of phylogeny, biogeography, and population genetics. For example, Zhou et al. reconstructed the panplastome of buckwheat and resolved phylogenetic relationships below the species level using single nucleotide variant (SNV) data [11]. Wang et al. assembled 316 plastomes of *Nelumbo nucifera*, constructed a panplastome genome atlas, mapped phylogeographic patterns, and resolved seven well-supported genetic clusters to describe the genetic diversity in this species [12]. Qihang Chen et al. studied the plastomes of East Asian peonies and used these data to resolve their evolutionary and domestication history [13]. Magdy et al. constructed the panplastome of *Capsicum* spp. using 321 individuals to clarify the taxonomic classification system among cultivated pepper lineages [14]. While recent studies [9] have used sequence data from the plastome to explore the genetic diversity of *Hemerocallis* species, they did not employ complete plastome data, nor did they examine the typically cultivated samples grown in China. Large-scale plastomic information is not yet available for *H. citrina*, yet the use of such data is an economical and rapid way to study intraspecific genomic diversity in relation to phylogeography, domestication history, germplasm preservation, and crop improvement.

In this study, we resequenced 65 *H. citrina* samples collected from 11 provinces in China and de novo assembled the complete plastomes. We combined these data with the complete plastome of *H. citrina* available in NCBI to make a comprehensive comparison among them. The objectives of this work were to (a) establish a reliable panplastome for *H. citrina*; (b) analyse codon usage bias and repetitive sequence features in the plastomes; (c) identify highly variable regions in the plastome to inform future marker development; (d) infer the population structure and genetic diversity of *H. citrina* to provide a basis for the identification of different *H. citrina* germplasm resources and the selection and breeding of improved varieties; and (e) reconstruct the maternal phylogenetic relationships of *H. citrina.*

## Materials and methods

### Plant material, plastome assembly and annotation

Sixty-five *H. citrina* samples were obtained from major production regions in China, including Hunan, Gansu, Shaanxi, Sichuan, Jiangsu, and Shanxi, and several field collections in Shanxi, as well as cultivars from each region. All *H. citrina* samples involved in this study were provided and preserved by the *H. citrina* Resource Nursery, College of Horticulture, Shanxi Agricultural University (Table S1). These samples were identified by the first author and further checked by Yunshan Wang (Institute of Horticulture, Shanxi Academy of Agricultural Sciences).Total DNA was extracted from each individual using a modified CTAB method [15]. Genomic DNA was sheared into 150 bp segments, and libraries were prepared with the TruSeq DNA Sample LT Prep kit (Illumina, San Diego, CA, US) as per the manufacturer’s guidelines and sequenced on the Illumina NovaSeq platform.

From the whole-genome shotgun sequencing data, plastome-specific sequences were extracted by aligning clean raw reads to the published *H. citrina* plastome (GenBank: NC_064967.1) using BWA v0.7.17 [16] and SAMtools v1.9 [17]. The extracted Illumina reads of all samples were de novo assembled using SPAdes 3.14 [18] and rendered into a circular molecule in Bandage 0.8.1 [19]. The two steps involved in the above refer to the pipelines described by He et al. [20]. The average assembly depth for each plastome surpassed 100×. In all obtained plastome sequences, the orientations of both the large single copy (LSC) and small single copy (SSC) regions were checked and manually adjusted to ensure maximum collinearity in the MEGA7 analyses for the convenience of downstream analyses [21]. The length and GC content of the different quadripartite structures of each plasmid were calculated using a custom Perl script, and the final data were processed using IBM SPSS Statistics 26 (SPSS Inc., Chicago, USA). Gene annotation of all plastomes was performed entirely using Geseq online software [22].

### Repeat sequences and codon usage bias

Codon usage bias in coding sequences of each plastome was analysed in codonW [23]. The results of relative synonymous codon usage (RSCU) were used to analyse the preference patterns of each sample relative to the entire set of *H. citrina* plastomes. The significance of differences in RSCU values was calculated using IBM SPSS Statistics 26 (SPSS Inc., Chicago, USA) using the Wilcoxon rank sum test. Tandem repeats in each plastome were identified using the Tandem Repeats Finder (TRF) [24] with default parameters. SSRs and repetitive sequences of all plastomes were analysed statistically using MISA.pl and Reputer [25]. One IR was ignored during repeat identification to avoid including these known repeat sequences in the final analyses.

### Population structure and phylogenetic analysis

All assembled complete plastome sequences were aligned by MAFFT 7 [26] with parameters set to default. The previously described aligned sequences were scanned using DnaSP 6 [27] to identify variant sites between different samples. The reference plastome used in this analysis was the *Hemerocallis citrina* cultivar ‘Datong Huanghua’. The positions of the genes with nucleotide variant sites were used for analysis and the types of variants were analysed in SnpEff [28]. Statistics and visualization of variant loci in different plastomes were performed in Microsoft Excel 2019 (Microsoft Corporation, USA). Based on the complete plastome dataset, population structure was inferred for all samples of *H. citrina* using ADMIXTURE 1.3 [29] with default settings and K values ranging from 1 to 10. The K value corresponding to the lowest CV value in the ADMIXTURE results was interpreted as the optimal K value. To perform phylogenetic analysis, an ML tree was reconstructed based on the complete plastome sequences using IQtree 2.1 [30] under the best model_BIC:UNREST+FO + R10 determined by the built-in ModelFinder Module.

### Haplotype and genetic diversity analyses

After the exclusion of indel sites based on corrected alignments, plastome haplotypes were determined by DNAsp 6 [27]. Haplotype networks were produced and plotted using Popart v1.7 [31] based on the median joining method to study the haplotype genealogical relationships of all samples. Image rendering and editing were performed in Adobe Illustrator software (Adobe Systems Incorporated, USA) on Popart output plots to facilitate easier interpretation of the results. Nucleotide diversity (π), haplotype diversity (Hd) and genetic differentiation (Fst) were calculated for the resolved lineages using DnaSP 6 [27] to assess and infer genetic diversity between and within different genetic clusters. Principal component analysis (PCA) was performed on all samples using TASSEL 5.0 [32], and the results obtained were visualized in Origin2021 (OriginLab Inc., Massachusetts, USA).

## Results

### Panplastome structure and organization

**Fig. 1:**
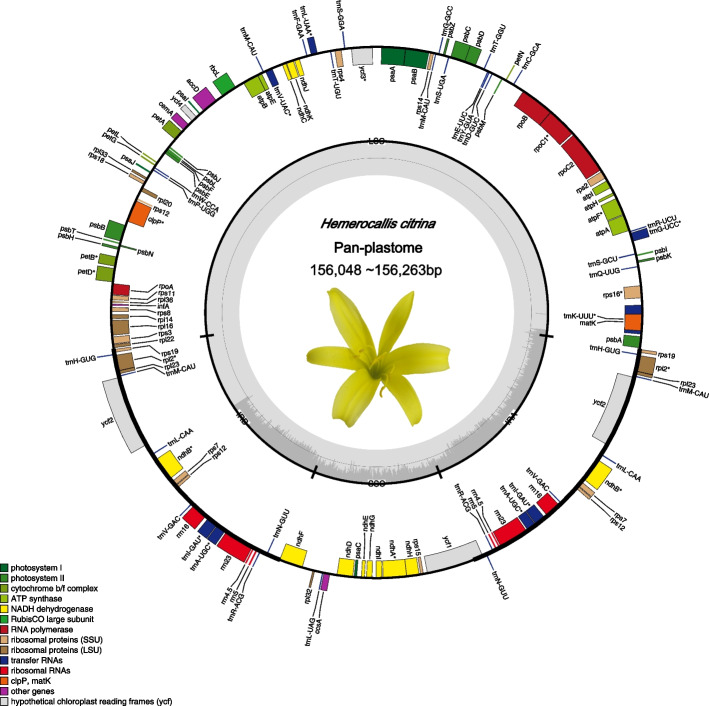
The panplastome of *H. citrina*. The inner genes of the outer circle are transcribed counterclockwise, while the outer genes are transcribed clockwise; genes with introns are marked with an asterisk (*ycf*3, *clp*P, and *rps*12 contain two introns, and all others contain one). Dark grey bars on the inner circle indicate GC content

In this study, 65 plastomes were successfully assembled using resequencing data. All assembled plastomes possessed a typical quadripartite structure, including an LSC, SSC, and a pair of inverted repeat regions (IRA and IRB). The full length of the plastomes of all the samples ranged from 156,048 bp to 156,263 bp, with an average length of 159,274.35 bp (the median length was 156,088 bp, Table S2, Fig. 1). The lengths of the IRs across samples were between 26,364 bp and 26,407 bp in length (x = 26,370.37 bp, median = 26,369 bp) and they were separated by an LSC between 84,492 bp and 84,798 bp in length, (x = 84,841.95 bp, median = 84,841 bp) and an SSC between 18,428 bp and 18,548 bp in length, (x = 18,513.37 bp, median = 18,507 bp, Table S2). The GC content of the complete plastomes ranged from 37.31 to 37.34% (x and median were both 37.34%). The GC content of the IR regions ranged from 42.86 to 42.89% (x and median were both 42.87%), the GC content of the LSC ranged from 35 to 35.08% (x and median were both 35.07%), and the SSC GC content was between 31.95 and 32.02% (x and median were both 31.97%, Table S2). Among the four genomic regions, the coefficients of variation for length and GC content were the highest in the SSC region (0.05%). The GC content of the IR regions was higher than that of LSC and SSC.

A total of 78 unique protein-coding genes (PCGs), 30 tRNA genes, and 4 rRNA genes were annotated and characterized in the plastomes of *H. citrina* (there are two copies each of 6 tRNA and 4 rRNA genes, Table 1, Fig. 1). The genes identified were distributed in the genome as follows: 80 genes in the LSC region, which consisted of 60 PCGs and 20 tRNAs; 14 genes in the SSC region, which consisted of 12 PCGs and one tRNA; and 17 genes in the IR regions, which included 6 PCGs, 4 rRNA genes, and 7 tRNAs. All genes in the IR regions had two copies (one in each IR) and were counted only once. The *rps*16, *atp*F, *tr*nG-UCC, *rpo*C1, *trn*K-UUU, *pet*B, *trn*L-UAA, *trn*V-UAC, and *pet*D genes were located in the large single-copy region containing one intron each, while *clp*P and *ycf*3 each contained two introns. In the SSC region, *ndh*A* contained one intron. In the IR regions, *ndh*B* *trn*A-UGC* *trn*I-GAU* *trn*L-UAG * and *rpl*2* each contained one intron. The *ycf1* gene spanned the junction of the SSC and IRA.

**Table 1:** Annotated genes in *H. citrina* plastomes

Gene Category	Functional Group	Gene Name
	Subunits of photosystem I	*psaA*, *psaB*, *psaC*, *psaI*, *psaJ*
	Subunits of photosystem II	*psbA*, *psbB*, *psbC*, *psbD*, *psbE*, *psbF*, *psbH*, *psbI*, *psbJ*, *psbK*, *psbT*, *psbL, psbZ*, *psbM*
	Small subunit of ribosome	*rps2*, *rps3*, *rps4*, *rps7* (2), *rps8*, *rps11*, *rps12*(2), *rps14*, *rps15*, *rps16*^a^, *rps18*, *rps19*(2),
	Large subunit of ribosome	*rpl2*^a^ (2), *rpl14*, *rpl16*, *rpl20*, *rpl22*, *rpl23* (2), *rpl32*, *rpl33*, *rpl36,*
	NADH dehydrogenase	*ndhA*^a^, *ndhB*^a^ (2), *ndhC*, *ndhD*, *ndhE*, *ndhF*, *ndhG*, *ndhH*, *ndhI*, *ndhJ*, *ndhK*
	Cytochrome b/f complex	*PetA*, *petB*^a^, *petD*^a^, *petG*, *petL*, *petN*
	Subunits of ATP synthase	*atpA*, *atpB*, *atpE*, *atpF*^a^, *atpH*, *atpI*
	RNA polymerase	*rpoA*, *rpoB*, *rpoC1*^a^, *rpoC2*
	Large subunit of Rubisco	*rbcL*
	Unknown function	*ycf1*, *ycf* 2(2), *ycf3*^b^, *ycf4*
	Cytochrome c biogenesis protein	*ccsA*
	Envelope membrane protein	*cemA*
	Subunit of ATP-dependent Clp	*clpP*^b^
	Translation initiation factor	*infA*
	Subunit of acetyl-CoA	*AccD*
	Maturase	*matK*
30 tRNA genes	Transfer RNA	*trnA-UGC*^a^ (2), *trnC-GCA*, *trnD-GUC*, *trnE-UUC*, *trnF-GAA*, *trnG-UCC*^a^, *trnG-GCC*, *trnH-GUC*, *trnH-GUG*, *trnI-CAU* (2), *trnI-GAU*^a^ (2), *trnK-UUU*^a^, *trnL-CAA* (2), *trnL-UAA*^a^, *trnL-UAG* ^a^, *trnM-CAU*, *trnN-GUU* (2), *trnP-UGG*, *trnQ-UUG*, *trnR-ACG* (2), *trnR-UCU*, *trnS-GCU*, *trnS-GGA*, *trnS-UGA*, *trnT-GGU*, *trnT-UGU*, *trnV-GAC*, *trnV-UAC*^a^, *trnW-CCA*, *trnY-GUA*,
4 rRNA genes	Ribosomal RNA	*4.5S rRNA* (2), *5S rRNA* (2), *16S rRNA* (2), *23S rRNA* (2)

^a^ Gene with one intron. ^b^ Gene with two introns. (2) Genes with two copies

### Repeat sequences

**Fig. 2:**
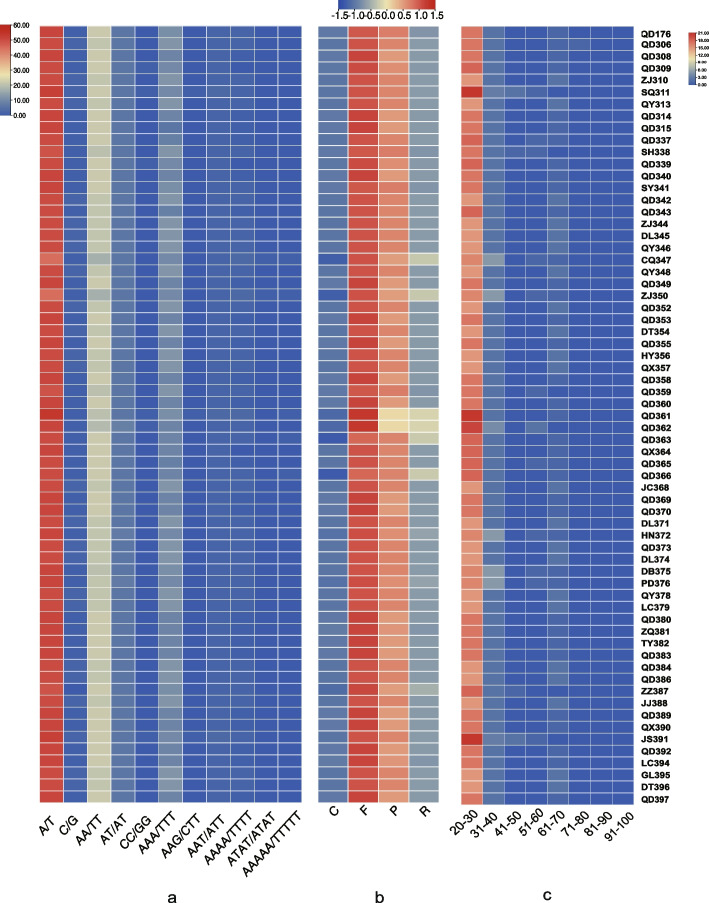
Abundance of repeat sequences annotated in *H. citrina*. **a** Eleven SSR motifs were detected; **b** interspersed repeat sequences (C, complement; F, forward; P, palindromic; R, reverse); **c** long tandem repeat sequences with lengths ranging from 20 bp to 100 bp in 10 bp increments

REPuter detected forward (F), palindromic (P), reverse (R) and complementary (C) repeats in the plastomes of all *H. citrina* samples, with the abundance of each repeat type varying by sample, with F repeats being the most abundant and C repeats being the least abundant (order of abundance F > P > R > C, Fig. 2). Repeat abundance also varied by sample, with samples QD361 and QD362 having fewer palindromic (P) repeat sequences than the other samples (10 repeats), while the same samples contained more reverse (R)-type repeats than the other samples (11 repeats compared to an average for all samples of 5.2). TRF was used to identify long tandem repeats of different lengths (20–100 bp). The 20–29 bp repeat category was the most abundant in all samples, with an average of 16.6 repeats. Samples SQ311, QD361, and JS391 had the highest abundance, with 21 such repeats. The 30–49 bp repeats were the second most abundant, with an average of 2.3 repeats per plastome. Among them, samples CQ347, ZJ350, HN372, DB375, and PD376 had the greatest number of repeats in this size category (with 5 repeats each). In the 50–69 bp category, only 5 *H. citrina* samples contained any repeats: SH338, ZZ387, SQ311, JS391 (all containing 1 repeat), and QD362 (containing 2). The 70–100 bp category contained no repeat sequences in any of the samples. The SSR search revealed 11 different SSR motifs, including 2 homopolymer SSRs, 3 dinucleotide SSRs, 3 trinucleotide SSRs, 2 tetranucleotide SSRs, and 1 pentanucleotide SSR, among which variants of the A/T type motifs were the most abundant. Among the samples, QD361 had the highest number of A/T SSRs with 54, and ZJ350 and CQ347 had the least with 45. The AA/TT motif was second in abundance to A/T, with an average of 21.3 per sample. Among the tetranucleotide SSRs, ATAT/ATAT was identified in only 2 samples, and the content of this type was only one. In total, 5 base repeats (AAAAA/TTTTT) were identified in 5 samples, all containing only one.

### Codon usage bias

Analyses of the plastome CDSs of all *H. citrina* samples in this study found variability among different lineages in codon usage. For instance, samples QD176, HN372, DL345, ZJ344, QD342, ZJ310, QY313, QD355, and QD397 used UUA in coding phenylalanine more frequently (*p* < 0.05), whereas coding of this amino acid by different codons was higher in all other samples (Fig. S1). Similarly, samples QD176, HN372, DL345, ZJ344, QD342, ZJ310, QY313, QD355, and QD397 also preferentially employed GCU in coding alanine, while most other samples employed this codon less (p < 0.05). In some cases, such as in the preference of AGA to code arginine, all samples were found to have a bias in the use of this codon over the other codons.

### Panplastome polymorphisms

To resolve differences in all plastomes at the nucleotide level, 65 newly assembled complete plastomes of *H. citrina* were used for the following analyses. All *H. citrina* samples were aligned with the reference genome *Hemerocallis citrina* cultivar ‘Datong Huanghua’, and a total of 848 nucleotide polymorphism sites were identified in the CDSs (coding DNA sequences), exons, introns, and IGSs (intergenic spacers). The most abundant variant type was SNVs with 590 (69.58%), followed by InDels with 157 (18.51%), and the least abundant were block substitutions with 101 (11.9%, Table S3). We further analysed these polymorphisms by the plastomic region and found that the CDSs contained 180 SNVs, 16 InDels, and 7 block substitutions; introns contained 37 SNVs, 19 InDels, and 3 block substitutions; and IGSs contained 373 SNVs, 122 InDels, and 91 block substitutions. The results showed that IGSs had the largest total number of polymorphisms (586, 69.1%), followed by CDSs (203, 23.94%), and the fewest were in introns (59, 6.96%) (Table S3).

**Fig. 3:**
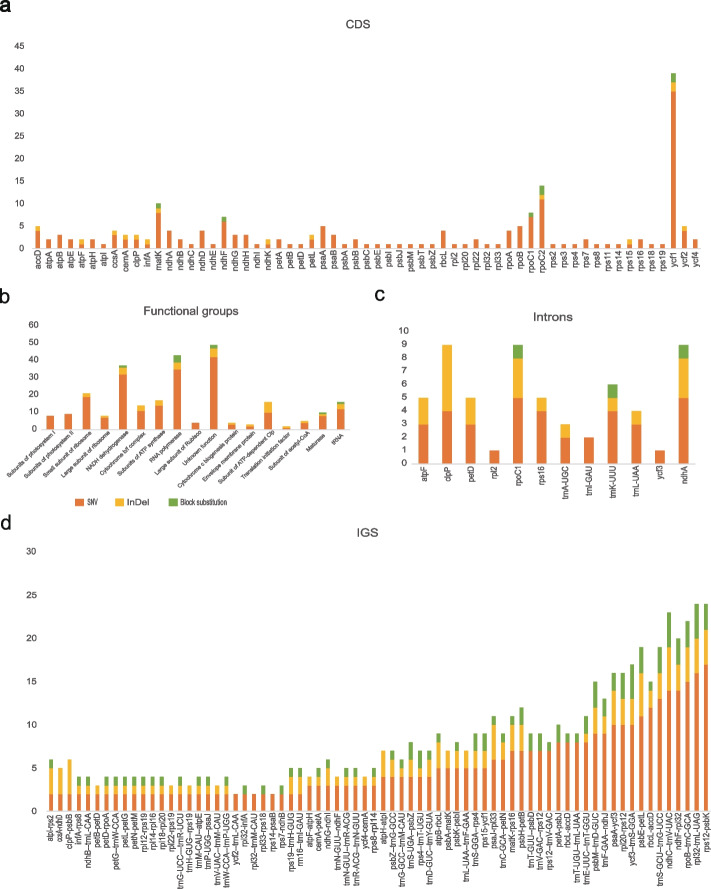
Variant locations categorized by genic position (**a** CDS, **b** functional grouping, **c** introns, **d** IGSs)

We analysed the polymorphisms in each gene in the panplastome with *ycf*1 (spanning the SSC and IR junction; 35 polymorphisms) having the most of any CDS region and *ndh*J, *pet*G, *psa*C, *psa*I, *pet*N, and *psb*D having the least, with no SNVs (Fig. 3a). Among introns, those in *clp*P, *rpo*C1, and *ndh*A contained the most polymorphic sites, all with nine. The *ndh*A gene is located in the SSC, and *clp*P and *rpo*C1 are located in the LSC. The introns in *ycf*3 located in the LSC contained the fewest polymorphisms, with just one (Fig. 3c). In the IGS regions, the sites with the highest concentration of polymorphisms were *ndh*F-*rpl*32 in the SSC (24 polymorphisms), *rpo*B--*trn*C-GCA in the SSC (24 polymorphisms), *rps*12-*psb*K in the LSC (24 polymorphisms), and *ndh*C--*trn*V-UAC in the LSC (23 polymorphisms) (Fig. 3d). Among the functional groups, NADH dehydrogenase contained the greatest number of polymorphisms (Fig. 3b). These results indicate that the LSC is the region evolving most rapidly within the plastomes of *H. citrina*.

### Population structure and genetic diversity

**Fig. 4:**
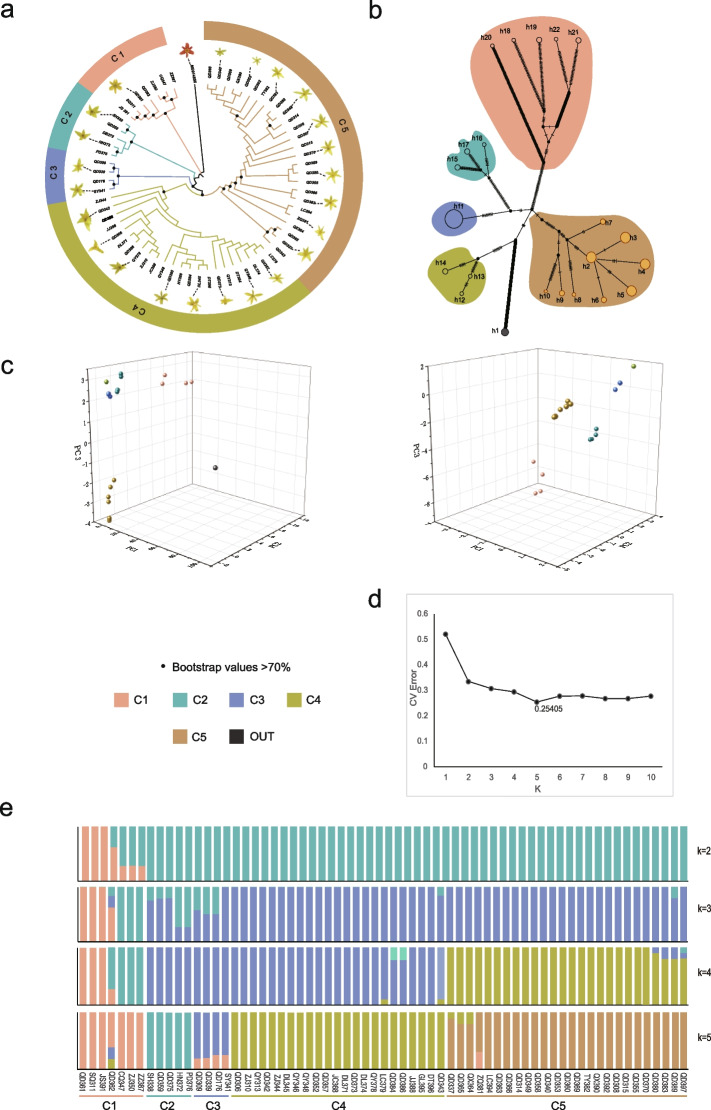
Population structure of *H. citrina* samples from China. **a** ML tree of 65 samples. **b** Haplotype network of *H. citrina* plastomes. Different colours represent different genetic clusters and correspond with the colour scheme throughout the figure. **c** PCA including *Hemerocallis fulva* (left) and excluding it (right). In both figures, the first three components are shown. **d** CV errors across a range of K values from 1 to 10 with the lowest CV error of 0.254 at K = 5. **e** Population structure bar plot at K = 2–5. K = 5, best matches the five major clusters of the phylogenetic analysis using the complete plastomes dataset

To elucidate the matrilineal relationships among different *H. citrina* samples, we used several methods to resolve population structure and to calculate genetic diversity among different groups of samples. Based on the completed plastomes, we analysed the population structure using ADMIXTURE. At K = 2, *H. citrina* samples in the first genetic cluster were not well distinguished; at K = 5 (the best-supported partitioning, Fig. 4d), the *H. citrina* samples were better resolved, although with some level of cross-cluster assignments. Sample QD362 in the C1 genetic cluster was found to have minor assignments to the C3 and C4 clusters. All samples of the C3 genetic cluster were found to have low-level assignments from the C1 genetic cluster, and ZQ381 in the C5 genetic cluster was found to have assignments to the C1 genetic cluster. The results of the network analysis (Fig. 4b) support the 5-cluster arrangement of *H. citrina* plastome diversity resolved via ADMIXTURE. Among the five genetic clusters, the vast majority of *H. citrina* from Qidong, Hunan Province, were assigned to C5. The other four genetic clusters all contained samples from different geographic locations (Table S1). The ML tree shows that the C1 lineage most recently split from the outgroup *Hemerocallis fulva* (Fig. 3a). Seven samples were classified in the C1 genetic cluster: QD361, SQ311, JS391, ZZ387, CQ347, ZJ350, and QD362. Sample QD362 was assigned partially to genetic Clusters C3 and C4, totalling 38%. The C1 genetic cluster contained individuals from Shanxi, Zhejiang, Hunan, Jiangsu, and Chongqing provinces. Four samples were assigned to the C4 genetic cluster, and each contained some C5 assignment, accounting for less than 25%.

We also employed three additional methods to analyse the population structure of all the samples: an ML phylogenetic tree constructed based on the complete plastid genome, PCA, and a median-joining network analysis using a matrix without gaps, all of which were consistent with the results of the ADMIXTURE analysis (Fig. 4a−c, e). Based on the results from all of these analyses, the *H. citrina* samples were divided into five genetic clusters. The analysis of the geography of the samples in each cluster revealed that not all individuals from the same region were grouped into a single genetic cluster (Table S4), and there was no clear pattern in terms of geography, except for Cluster C5, which was mostly composed of samples from Qidong County, Hunan Province. The three samples from Zhejiang were divided into two genetic clusters, C1 and C4, while the individuals from Hunan were divided into five genetic clusters, but most of the individuals from Qidong, Hunan, were assigned to the C5 genetic cluster. Datong *H. citrina* from Shanxi was assigned to genetic Cluster C3 along with samples from Dali*,* Shaanxi, Qingyang, and Gansu (Fig. 4a−c, e).

**Fig. 5:**
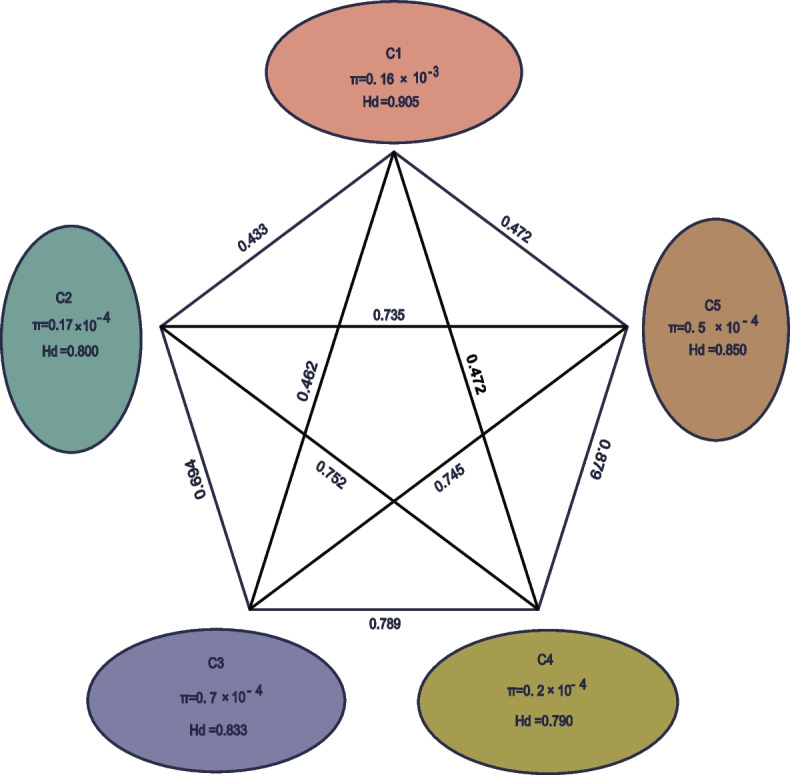
Genetic diversity and differentiation of five genetic clusters of *H. citrina*. Numbers above lines connecting two bubbles represent pairwise Fst calculated between respective genetic clusters

Based on the complete plastome alignment, we constructed a median-joining network, by which 65 *H. citrina* samples were divided into 21 haplotypes. Cluster C5 contained nine haplotypes, the highest number of any genetic cluster. Among the five genetic clusters, the highest haplotype diversity (Hd) was found in C1 (Hd = 0.905, π = 0.16 × 10^−3^), followed by C5 (Hd = 0.85, π = 5 × 10^−4^), C3 (Hd = 0.8333, π = 0.7 × 10^−3^), C2 (Hd = 0.8, π = 0.17 × 10^−3^), and C4 (Hd = 0.79, π = 0.2 × 10^−3^) (Fig. 5). We assessed genetic divergence by calculating Fst values between each genetic cluster. As shown in Fig. 5, the degree of population differentiation (Fst) between C5 and C4 was the greatest, followed by C3 and C4 (Fst = 0.789), C4 and C2 (Fst = 0.752), and C5 and C2 (Fst = 0.735), with the degree of population differentiation between C1 and C2 being the lowest (Fst = 0.433) (Fig. 5).

## Discussion

### Hypervariable regions across the *H. citrina* panplastome

In this study, 65 *H. citrina* samples were resequenced, and a panplastome was constructed from the sequence data. Plastome analysis of all samples revealed that the plastomes of *H. citrina*, similar to those of other higher plants, are also highly conserved in terms of quadripartite structure, length, gene order, and GC content [33, 34]. In terms of tetrad length variation, the coefficient of variation in the length of the SSC region between samples of *H. citrina* was greater than that of the LSC and IR regions, which is the result of ongoing contraction and expansion of the SSC region over time (Table S2). We further analysed the IRa/SSC junctions (Fig. S2) and found that *ycf1* was of different lengths in the SSC in some genetic clusters. This suggests that the junctions on either side of the SSC region could be used as highly variable sites for the development of molecular markers. The GC content of the IR regions is higher than that of the LSC and SSC regions in the plastomes of *H. citrina* [35, 36].

**Fig. 6:**
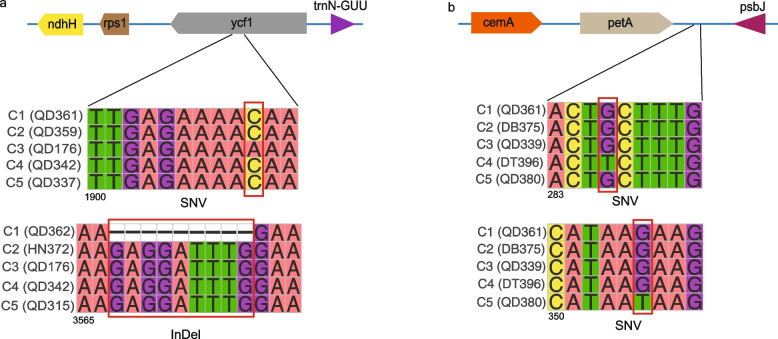
Examples of variable sites. (**a**) SNV and InDel sites in the *ycf*1 gene in samples from different genetic clusters of *H. citrina.* (**b**) SNV sites in the *pet*A-*psb*J IGS. (1900, 283, 3565 and 350 represent the positions of the sequences where the corresponding bases are located)

Despite structural and genic conservation in the plastomes, numerous nucleotide variations were found across the *H. citrina* panplastome. These nucleotide variants can be used in the development of molecular markers for the identification of *H. citrina* maternal lineages [37]. Among the variant identification results in all CDSs, a large number of variants were found in *ycf*1, which noticeably outnumbered those in other genes. This is most likely related to the location of *ycf*1, as it is located in a position spanning the IRA and SSC boundary and is more affected by SSC contraction and expansion than other genes. This is similar to previous studies of the Asian lotus panplastome [12]. For example, when we further analysed the variants of *ycf*1, we found that there was a single fixed base variant of A > C at site 1910 of *ycf*1, which could be used to distinguish samples from the C3 genetic cluster from the other genetic clusters and may serve as a locus for molecular identification of samples from the C3 genetic cluster **(**Fig. 6a). The *ycf*1 gene also contains a unique indel at 3567–3515 that distinguishes C1 from other clusters. **(**Fig. 6a). In addition to *ycf*1, more informative nucleotide variants were found in the *rpo*C2 and *mat*K genes from samples in this study. Among the CDSs, block substitutions were found only in four genes, namely, *rpo*C2, *rpo*C1, *ndh*F, and *mat*K, and did not alter the protein sequences through frameshift mutation. Similarly, the number of InDels found in CDSs was low, with only 13 genes containing such polymorphisms. The number of InDels found in noncoding regions was higher than in other regions, following the pattern found in numerous plastomes of other plant species [38]. In contrast, the IGS regions and introns were rich in nucleotide variants compared to the CDSs. The 10 most polymorphic IGS regions were *psa*A-*ycf*3, *rpl*20-*rps*12, *ycf*3--*trn*S-GGA, *psb*E-*pet*L, *trn*S-GCU--*trn*G-UCC, *ndh*F-*rpl*32, *rpo*B--*trn*C-GCA, *ndh*C--*trn*V-UAC, *rpl*32-*trn*L-UAG, and *rps*12-*psb*K. For example, nucleotide variants at sites 286 and 305 of the *petA-psbJ* IGS could be used to distinguish samples from the C4 and C5 genetic clusters from the others (Fig. 6b). The results of these polymorphic site assays indicate that the proportion of variation in coding regions is much lower than that in noncoding regions, similar to previous results in the panplastome of *Fagopyrum* [37, 39, 40]. This is also in keeping with previous studies related to the rate of evolution by selection on coding and noncoding regions and the importance of the more highly variable noncoding regions for phylogenetic studies in plants [37, 39, 40]. Variant analyses from different genomic regions of the plastome revealed that the variants in the LSC and SSC regions were relatively higher than those in the IR region, which is consistent with most plastome studies conducted thus far [35–37]. As more panplastomes are completed, the high variability in IGSs will be confirmed, and detailed patterns of intraspecific molecular evolution will be resolved. Thus, all variants found in the IGSs during subsequent systematic studies can be used to improve the resolution of intraspecific studies with the currently generated panplastome.

### Phylogenetic relationships and population structure

With the rapid development and refinement of sequencing technologies and analytical methods, more plastomic data have been successfully used in phylogenetic studies [41, 42]. We collected 65 samples of *H. citrina* from seven major production areas in China, namely, Shanxi, Shaanxi, Hunan, Gansu, Chongqing, Zhejiang, and Sichuan. We resequenced these 65 samples and successfully assembled 65 complete plastomes, from which we reconstructed a well-supported phylogenetic tree within *H. citrina*. This phylogenetic tree divided all the samples into five well-supported lineages, among which genetic Clusters C1 and C2 diverged earliest. In addition, the various population structure analyses used here indicated that ZJ350 from Zhejiang, CQ347 from Chongqing, JS391 and JS311 from Jiangsu, ZZ387 from Zezhou, southwestern Shanxi, and QD362 and QD361 from Qidong, Hunan were grouped into a single genetic cluster (C1) and that despite the differing geographic origins of the samples, the samples were noted as being both ornamental and edible. Although there are no genes directly regulating flower colour in the plastome, our results suggest that these samples are all related through maternal descent. Our findings are similar to the results of the CSoT markers utilized by Zheng et al. [43] on the genetic diversity of *H. citrina* genetic resources in China, which clustered the orange–red flowering samples into a single group. In addition, the phylogenetic tree also showed that QX357 from Quxian, Sichuan Province, was clustered with DT396 from Datong, Shanxi Province, GL395 from Guangling, Shanxi Province, and GS348 from Qingyang, Gansu Province, where the clustering of QX357 and DT395 were similar to the results of cluster analysis of *H. citrina* germplasm resources based on leaf, flower, and root traits used by Liu, Z. et al. [44]. The results reported here indicate that the study of species affinities based on whole plastome data is reliable at multiple taxonomic levels, including the intraspecific level. This is the first panplastome-based study of the genealogical history in *H. citrina*, which not only adds to our understanding of the intraspecific relationships of *H. citrina* but also provides geographic locations that should yield new genetic diversity upon renewed exploration, especially in Hunan where very high haplotypic diversity was noted.

## Conclusions

We constructed a panplastome of *H. citrina* using 65 newly assembled plastid genomes. We found that the panplastome of *H. citrina* was highly conserved in terms of plastome length, gene, and GC content, as it is in other species, yet numerous informative polymorphisms were present throughout the plastome for the study of maternal genetic diversity. In this study, we systematically analysed the nucleotide variability in the panplastome of *H. citrina*, and in addition to the previously reported regions of high variability, we identified several new regions, such as *ycf*1 and *rpo*C2. In this study, based on the complete plastome dataset and using 3 different methods of population structure analysis, the 65 *H. citrina* samples were categorized into 5 genetic clusters (C1, C2, C3, C4, and C5). By mapping cultivar attributes and geographic location onto the population structure, it was found that C5 from Qidong, Hunan Province, was the only genetic cluster with a narrow geographic distribution and that all *H. citrina* with ornamental and edible properties clustered into a single early diverging lineage (C1). Therefore, although the plastome does not contain genes regulating flower colour directly, it can be used to resolve maternal relationships that in turn correspond to economically important traits. Overall, the data presented here can be employed in marker-assisted breeding programs and in the identification and preservation of wild and cultivated germplasm.

### Supplementary Information

**Additional file 1.**

## Data Availability

The complete plastomes in this study were submitted to the NCBI website (https://www.ncbi.nlm.nih.gov/) under accession OR497837. All other data and material analyzed in the current study are included in the manuscript and the supplementary information.
