# Supplementary material for: The pan-plastome of Hemerocallis citrina reveals new insights into the genetic diversity and cultivation history of an economically important food plant
Source: BMC Plant Biol. 2024 Jan 11;24:44. doi: 10.1186/s12870-023-04668-z (PMC10782787; doi:10.1186/s12870-023-04668-z)
Supplement: Supplementary file 1 — Additional file 1. [file 12870_2023_4668_MOESM1_ESM.docx]

**Table S1** Information on 65 *H.* *citrina* samples.

| **Accession** | **Sample Source** | **Specimen Information** | **Sample Characteristics** | **Genetic Cluster** |
| --- | --- | --- | --- | --- |
| ZJ310 | Xianju County, Zhejiang Province; XianJuHua | SXAU001310 | Precocious | C4 |
| QD315 | Qidong County Hunan Province | SXAU001315 | Precocious; Early Flower | C5 |
| QY313 | Qingyang City, Gansu Province Xiaohuahuanghua | SXAU001313 | Precocious | C4 |
| QD176 | Qidong County Hunan Province Siyuehua | SXAU001176 | Precocious | C3 |
| QD349 | Qidong County Hunan Province; Siyuebai | SXAU001349 | Precocious | C5 |
| QX364 | Quxian County, Sichuan Province Wupinghua | SXAU001364 | Precocious | C5 |
| SQ311 | Suqian City, Jiangsu Province HY | SXAU001311 | Precocious; Ornamental and edible | C1 |
| QD337 | Qidong County Hunan Province | SXAU001337 | Precocious; Ornamental and edible | C5 |
| SH338 | Shanghai;Shangyingshuangjihua | SXAU001338 | Precocious; Ornamental and edible | C2 |
| CQ347 | Chongqing | SXAU001347 | Precocious; Ornamental and edible | C1 |
| QD359 | Qidong County Hunan Province AnminNo.1 | SXAU001359 | Precocious; Ornamental and edible | C2 |
| QD365 | Qidong County Hunan Province; Anmin | SXAU001365 | Precocious;Ornamental and edible | C5 |
| QD308 | Hunan Province Anmin;TangHuangHua | SXAU001308 | Moderate maturity | C5 |
| QD309 | Qidong County Hunan Province CaiQiaoHua | SXAU001309 | Moderate maturity | C3 |
| QD314 | Qidong County Hunan Province | SXAU001314 | Moderate maturity | C5 |
| QD339 | Qidong ,County Hunan ProvinceQizhenbianyi | SXAU001339 | Moderate maturity | C3 |
| QD340 | Qidong County Hunan ProvinceQizhen | SXAU001340 | Moderate maturity | C1 |
| SY341 | Shaoyang City, Hunan ProvinceChangzuizihua | SXAU001341 | Moderate maturity | C3 |
| QD342 | Qidong ,County Hunan Province; Daojianhua | SXAU001342 | Moderate maturity | C4 |
| ZJ344 | Jinyun County, Zhejiang Province; Panlonghua | SXAU001344 | Moderate maturity | C4 |
| DL345 | Dali County, Shaanxi Province; Shayuanjinzhen | SXAU001345 | Moderate maturity | C4 |
| QY346 | Qingyang City, Gansu Province | SXAU001346 | Moderate maturity | C4 |
| QY348 | Qingyang City, Gansu ProvinceXianhuanghua | SXAU001348 | Moderate maturity | C4 |
| QD353 | Qidong County Hunan Province Baihua | SXAU001353 | Moderate maturity | C5 |
| DT354 | Datong City, Shanxi Province | SXAU001354 | Moderate maturity | C4 |
| QD355 | Qidong County Hunan Province Mengzihua | SXAU001355 | Moderate maturity | C5 |
| HY356 | Huaiyang, Henan Provincehuaiyanghua | SXAU001356 | Moderate maturity | C4 |
| QX357 | Quxian County, Sichuan Province | SXAU001358 | Moderate maturity | C4 |
| QD358 | Qidong County Hunan Province | SXAU001360 | Moderate maturity | C5 |
| QD360 | Qidong County Hunan Province; Xinbaihua | SXAU001366 | Moderate maturity | C5 |
| QD366 | Qidong County Hunan Province; Mengzihua Bianyi | SXAU001343 | Moderate maturity | C5 |
| QD343 | Qidong County Hunan Province | SXAU001350 | Moderate maturity; Ornamental and edible | C5 |
| ZJ350 | Longyou County, Zhejiang Province; Longyouhonghuacai | SXAU001361 | Ornamental and edible | C1 |
| QD361 | Qidong County Hunan Province; Zhongmudan | SXAU001306 | Ornamental and edible; | C1 |
| QD306 | QidongCounty,Hunan Province | SXAU001352 | Late maturity | C4 |
| QD352 | Qidong County Hunan Province Xiyezihua | SXAU001362 | Late maturity | C4 |
| QD362 | Qidong County Hunan Province Anmin | SXAU001363 | Late maturity | C1 |
| QD363 | Qidong County Hunan Province;Qizhen Bianzhong | SXAU001378 | Late maturity | C5 |
| QY378 | Qingyang City, Gansu Province;Gansuhuanghua | SXAU001376 | unrecorded | C4 |
| PD376 | Pingding County, Shanxi ProvincePingdingNo.1 | SXAU001371 | unrecorded | C2 |
| DL371 | Dali County, Shaanxi Province;Shaaxihuanhua | SXAU001389 | unrecorded | C4 |
| QD389 | Qidong County Hunan Province;Baihua | SXAU001368 | unrecorded | C5 |
| JC368 | Jiaocheng County, Shanxi Province | SXAU001396 | unrecorded | C4 |
| DT396 | Datong City, Shanxi Province | SXAU001374 | unrecorded | C5 |
| DL374 | Dali County, Shaanxi ProvinceShawanhua |  | unrecorded | C4 |
| GL395 | Guangling County, Shanxi Province; GuanglingNo2 | SXAU001395 | unrecorded | C4 |
| QD383 | Qidong County Hunan Province;Mengzihua | SXAU001383 | unrecorded | C5 |
| QD384 | Qidong County Hunan Province;4Siyuehua | SXAU001384 | unrecorded | C4 |
| QD392 | Qidong County Hunan Province | SXAU001392 | unrecorded | C5 |
| QD386 | Qidong County Hunan Province | SXAU001386 | unrecorded | C4 |
| LC379 | Shanxi Province; LichengNo2 | SXAU001379 | unrecorded | C4 |
| LC394 | Shanxi Province; LichengNo5 | SXAU001394 | unrecorded | C5 |
| HN372 | Henanhuanghua | SXAU001372 | unrecorded | C2 |
| QD380 | Qidong County Hunan Province,Caichaohua | SXAU001380 | unrecorded | C5 |
| QD373 | Qidong County Hunan Province Xiyezihua | SXAU001373 | unrecorded | C4 |
| QX390 | Quxian County, Sichuan Province Quxianbianzhong | SXAU001390 | unrecorded | C5 |
| JS391 | Shuyang County, Jiangsu Province | SXAU001391 | unrecorded | C1 |
| DB375 | Dongbeihuanghua | SXAU001375 | unrecorded | C2 |
| QD397 | Qidong County Hunan Province, Qiezihua | SXAU001397 | unrecorded | C5 |
| ZZ387 | Jincheng City, Shanxi Province | SXAU001387 | unrecorded | C1 |
| QD370 | Qidong County Hunan Province Hunanhuanghua | SXAU001370 | unrecorded | C5 |
| JJ388 | Jiaocheng County, Shanxi Province Jiaocheng No3 | SXAU001388 | unrecorded | C4 |
| ZQ381 | Zuoquan County, Shanxi Province ZuoquanNo1 | SXAU001381 | unrecorded | C5 |
| QD369 | Qidong County Hunan Province Chonglihua | SXAU001369 | unrecorded | C5 |
| TY382 | Taiyuan City, Shanxi Province TaiyuanNo2 | SXAU001382 | unrecorded | C5 |

**Table S2.** Characteristics of 65 newly assembled plastomes

| **Length (bp)** | | | | | | | **GC content (%)** | | | | | |
| --- | --- | --- | --- | --- | --- | --- | --- | --- | --- | --- | --- | --- |
|  | Minimum | Maximum | Median | ‾x | σ | CV | Minimum | Maximum | Median | ‾x | σ | CV |
| LSC | 84992 | 84798 | 84841 | 84841.95 | 28.91 | 0.03% | 35 | 35.08 | 35.07 | 35.07 | 0.014 | 0.04% |
| IRA/IRB | 26364 | 26407 | 26369 | 26370.37 | 6.14 | 0.02% | 42.86 | 42.89 | 42.87 | 42.87 | 0.007 | 0.02% |
| SSC | 18428 | 18548 | 18507 | 18513.37 | 13.93 | 0.08% | 31.94 | 32.02 | 31.97 | 31.97 | 0.017 | 0.05% |
| Total | 156048 | 156263 | 156088 | 159274.35 | 38.24 | 0.03% | 37.31 | 37.34 | 37.34 | 37.34 | 0.007 | 0.02% |

**Table S3** Number of variants among 65 *H.* *citrina* accessions.

| Variants | Total | Region | | | Location | | |
| --- | --- | --- | --- | --- | --- | --- | --- |
|  |  | LSC | SSC | IRA/B | CDS | Intron | IGS |
| SNV | 590 | 445 | 85 | 30 | 180 | 37 | 373 |
| InDel | 157 | 132 | 11 | 7 | 16 | 19 | 122 |
| Block substitution | 101 | 82 | 7 | 6 | 7 | 3 | 91 |
| Total | 848 | 659 | 103 | 43 | 203 | 59 | 586 |

**Table S4** Number of variants /Length of the region

| Variants | Region | | |
| --- | --- | --- | --- |
|  | LSC | SSC | IRA/B |
| SNV | 0.0052^*^ | 0.0010 | 0.0003 |
| InDel | 0.0050^*^ | 0.0004 | 0.0002 |
| Block substitution | 0.0044^*^ | 0.0003 | 0.0003 |
| Total | 0.0041^*^ | 0.0006 | 0.0002 |

*Represents p < 0.05.

**Table S5** Haplotype diversity by province

| Province/Region | Number of haplotypes |
| --- | --- |
| Hunan | 14 |
| Gansu | 1 |
| Chongqing | 1 |
| Shanxi | 5 |
| Shanghai | 1 |
| Zhejiang | 2 |
| Henan | 2 |
| Shaanxi | 1 |
| Jiangsu | 1 |
| Sichuan | 3 |
| Dongbei | 1 |

**Table S6** Haplotypes among65 *H.* *citrina* samples.

| Haplotype | accession | | | | | | | | | | | |  |
| --- | --- | --- | --- | --- | --- | --- | --- | --- | --- | --- | --- | --- | --- |
| H1 | MG914655 |  |  |  |  |  |  |  |  |  |  |  | |
| H2 | QD397 | QD389 | QD383 | QD380 | QD370 | QD355 | QD315 |  |  |  |  |  | |
| H3 | QD308 |  |  |  |  |  |  |  |  |  |  |  | |
| H4 | QD392 | QX390 | hh382 | hh369 | QD360 | QD353 | QD340 |  |  |  |  |  | |
| H5 | QD358 | QD349 | QD314 |  |  |  |  |  |  |  |  |  | |
| H6 | QD366 | QD363 |  |  |  |  |  |  |  |  |  |  | |
| H7 | LC394 | TY381 |  |  |  |  |  |  |  |  |  |  | |
| H8 | QX364 |  |  |  |  |  |  |  |  |  |  |  | |
| H9 | QD365 | QD337 |  |  |  |  |  |  |  |  |  |  | |
| H10 | QD343 |  |  |  |  |  |  |  |  |  |  |  | |
|  |  |  |  |  |  |  |  |  |  |  |  |  | |
| H11 | DT396 | QD386 | QD384 | QY378 | QD373 | JC368 | HY356 | QD352 | QY346 | ZJ344 | QY313 | QD306 | |
|  | GL395 | JJ388 | LC379 | DL374 | DL371 | QX357 | DT354 | QY348 | DT345 | QD342 | ZJ310 |  | |
| H12 | SY341 |  |  |  |  |  |  |  |  |  |  |  | |
| H13 | QD176 |  |  |  |  |  |  |  |  |  |  |  | |
| H14 | QD339 | QD309 |  |  |  |  |  |  |  |  |  |  | |
| H15 | QD376 | HN372 |  |  |  |  |  |  |  |  |  |  | |
| H16 | DB375 |  |  |  |  |  |  |  |  |  |  |  | |
| H17 | QD359 | SH338 |  |  |  |  |  |  |  |  |  |  | |
| H18 | ZZ387 |  |  |  |  |  |  |  |  |  |  |  | |
| H19 | ZJ350 | CQ347 |  |  |  |  |  |  |  |  |  |  | |
| H20 | QD362 |  |  |  |  |  |  |  |  |  |  |  | |
| H21 | JS391 | SQ311 |  |  |  |  |  |  |  |  |  |  | |
| H22 | QD361 |  |  |  |  |  |  |  |  |  |  |  | |

**
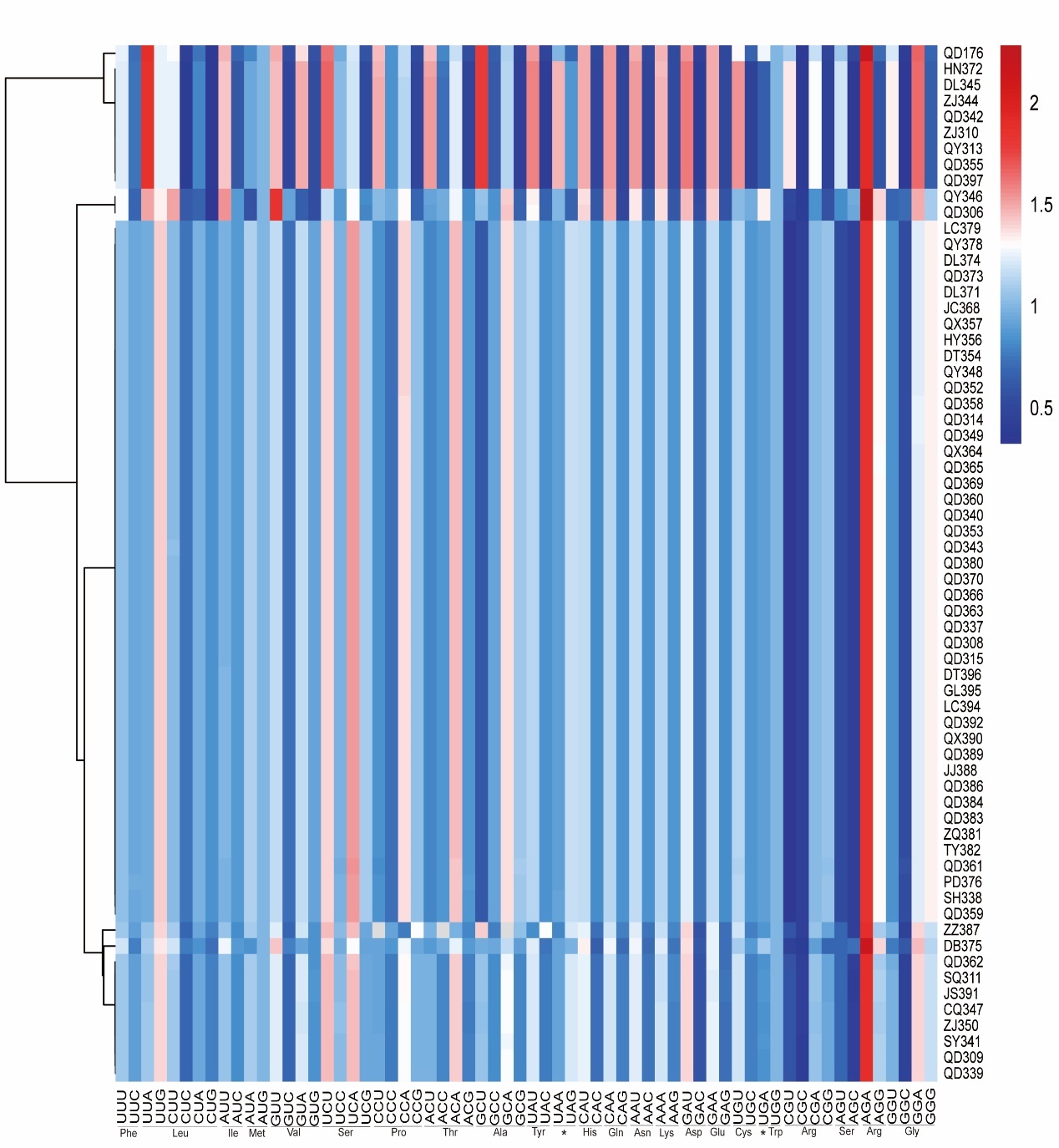
**

**Fig.S1** Heatmap of the mean RSCU values for 65 *H. citrina* samples**.**


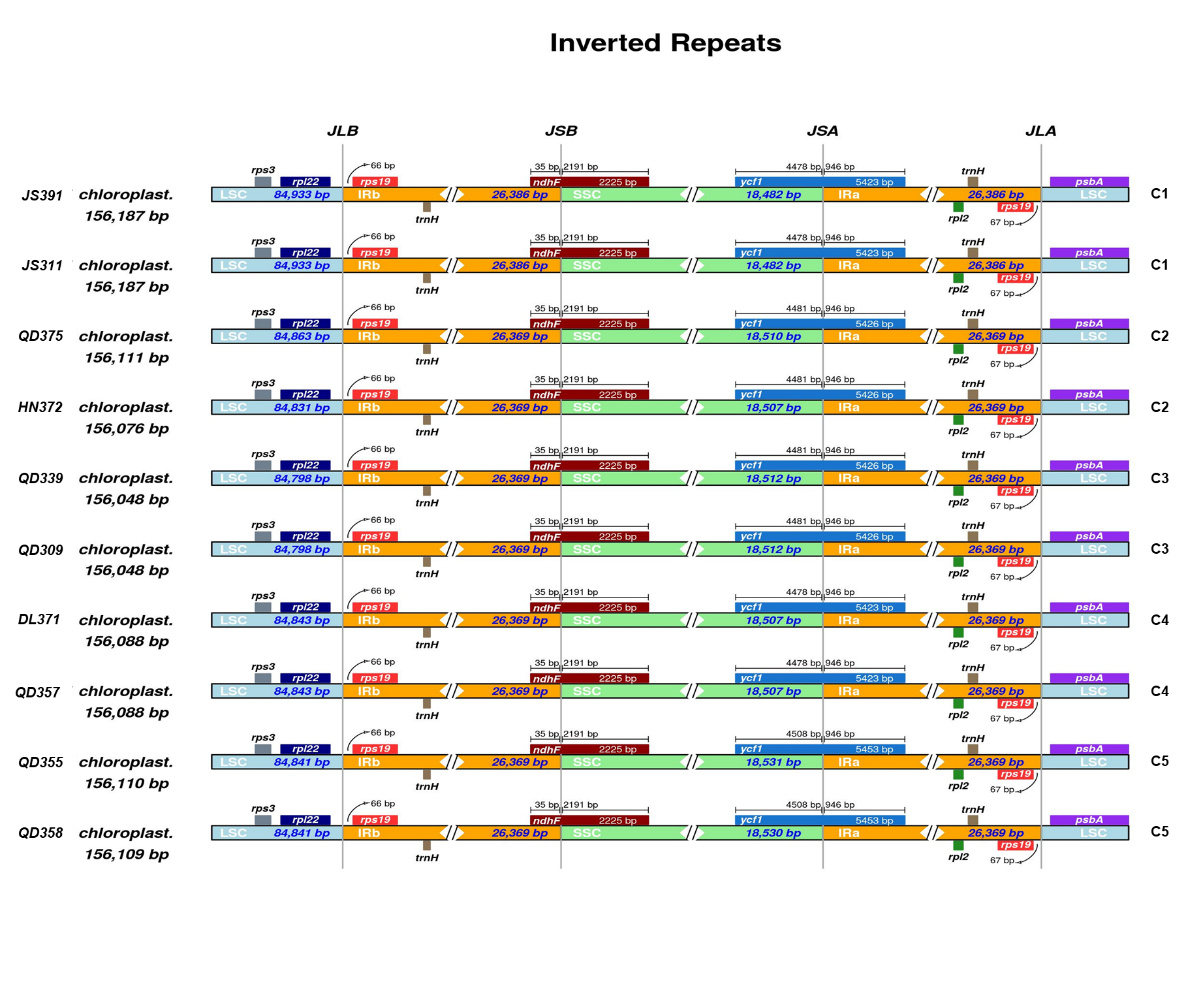


**Fig.S2** Structure of inverted repeat regions (IRs) in the plastids of *H. citrina*. Two samples were selected for each genetic cluster.


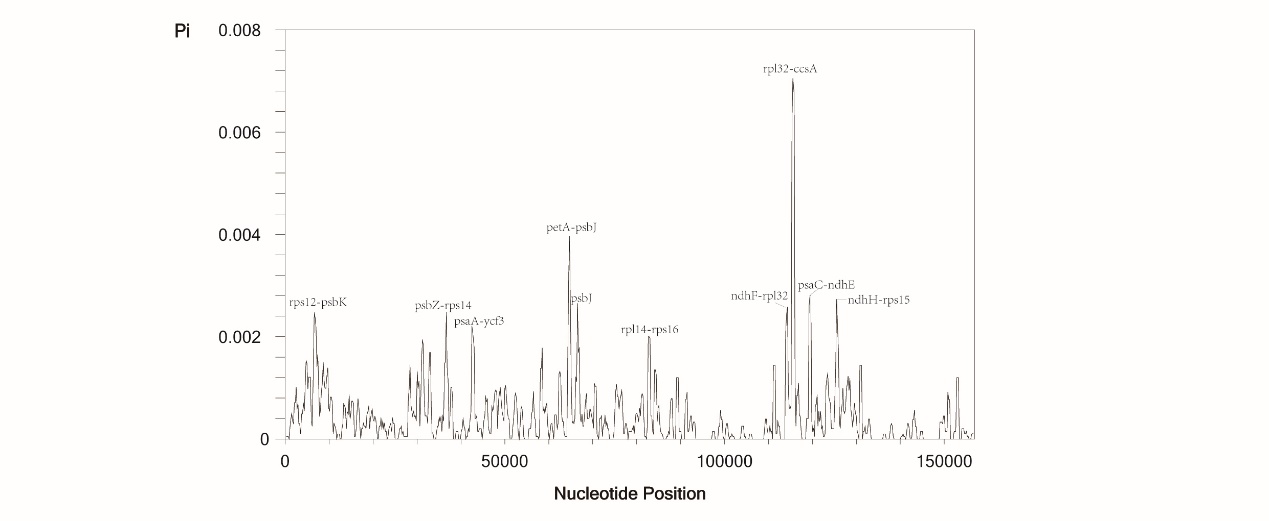


Fig S3 Highly polymorphic regions across the alignment of 65 *H. citrina* samples
